# Antioxidant Peptides from *Skipjack tuna*: Ameliorate Function on Cigarette Smoke Extract-Induced COPD in Cell Model by Targeting Oxidative Stress, Inflammation and Apoptosis

**DOI:** 10.3390/md24040140

**Published:** 2026-04-16

**Authors:** Yu-Hui Zeng, Yang-Yan Jin, Yan Sheng, Chang-Feng Chi, Bin Wang

**Affiliations:** 1 National and Provincial Joint Laboratory of Exploration, Utilization of Marine Aquatic Genetic Re-Sources, National Engineering Research Center of Marine Facilities Aquaculture, School of Marine Science and Technology, Zhejiang Ocean University, Zhoushan 316022, China; 2 Zhejiang Provincial Engineering Technology Research Center of Marine Biomedical Products, School of Food and Pharmacy, Zhejiang Ocean University, Zhoushan 316022, China

**Keywords:** *Skipjack tuna*, antioxidant peptide, cigarette smoke extract, chronic obstructive pulmonary disease (COPD), oxidative stress, inflammatory response

## Abstract

Antioxidant peptides show significant activity and can be developed into functional foods for treating chronic diseases. Cigarette smoke components can cause damage or even apoptosis of lung cells, eventually leading to chronic lung diseases. Therefore, this study aimed to investigate the protective effects and mechanisms of *Skipjack tuna* peptides against in vitro cigarette smoke extract (CSE)-induced chronic obstructive pulmonary disease (COPD). The results demonstrated that tuna peptides DVGRG (S1), PHPR (S5), GRVPR (S6), and SVTEV (S7) significantly enhanced the activities of SOD, CAT, and GSH-Px by upregulating the mRNA transcription levels of Keap1 and Nrf2, consequently reducing ROS and MDA levels in CSE-induced COPD model of MLE-12 cells. Molecular docking analysis revealed that S1, S6, and S7 competitively inhibited the Keap1-Nrf2 interaction by binding to the Kelch domain of Keap1, whereas S5 operated through a non-competitive mechanism. These peptides also downregulated p65 mRNA expression and upregulated IκBα mRNA expression, leading to a significant reduction in inflammatory cytokines of IL-1β, IL-6, and TNF-α, thereby alleviating inflammatory responses. Furthermore, these peptides significantly inhibited CSE-induced apoptosis by restoring mitochondrial membrane potential and upregulating the Bcl-2/Bax ratio. Additionally, S1, S5, S6, and S7 promoted MLE-12 cell migration in a concentration-dependent manner, suggesting a role in lung epithelial repair and regeneration. In conclusion, tuna peptides S1, S5, S6, and S7 exert antioxidant, anti-inflammatory, anti-apoptotic, and cell migration-promoting effects through the regulation of the Keap1/Nrf2 and NF-κB signaling pathways, as well as Bcl-2/Bax apoptotic balance, providing a promising strategy for mitigating CSE-induced lung injury.

## 1. Introduction

Bioactive peptides produced from fish, shellfish, algae, and their processing by-products show a variety of significant biological activities, such as antioxidant, anticoagulant, antihypertensive, anti-inflammatory, anti-cancer, antimicrobial, and other properties [1,2]. The differences in the activities of peptides mainly lie in molecular characteristics, such as molecular size, peptide sequence, and three-dimensional structure [3]. Recently, marine antioxidant peptides have attracted great interest due to their latent possibility to treat chronic diseases [3,4]. For example, oyster peptides enhance cellular antioxidant defense by activating the Nrf2-Keap1 pathway, while antioxidant peptides from bonito fish roe exhibit potent ROS-scavenging activity [5]. Moreover, high Fischer ratio oligopeptides (HFOPs-AK) from Antarctic krill have been shown to regulate oxidative stress and inflammation by modulating the Nrf2/NF-κB (IκBα) pathway [6]. Therefore, the development of bioactive peptides using marine foods and their processing by-products not only increases the added value of marine foods, reduces resource waste and protects the environment, but also may provide high-quality functional foods, and even safer and more effective alternatives to existing drugs. Despite these advancements, the potential of marine-derived peptides in the treatment of chronic obstructive pulmonary disease (COPD) has not been thoroughly investigated.

Smoking, a major global public health challenge, is the most significant preventable risk factor for chronic obstructive pulmonary disease (COPD) [7]. Cigarette smoke (CS) contains over 7000 chemicals, including highly reactive free radicals and reactive oxygen species (ROS), which directly induce pulmonary oxidative stress. Components such as benzoquinones can penetrate the blood–gas barrier and continuously generate superoxide radicals via quinone redox cycling, leading to molecular damage such as DNA strand breaks, lipid peroxidation, and protein oxidation [8]. Studies indicate that smokers exhibit significantly higher oxidative stress levels, and COPD patients who smoke demonstrate lower endogenous antioxidant levels (e.g., superoxide dismutase (SOD), reduced glutathione (GSH) and glutathione peroxidase (GPx)) compared to non-smoking COPD patients, suggesting that smoking exacerbates oxidative–antioxidant imbalance in COPD [9,10]. These oxidative insults further activate the NF-κB inflammatory pathway, promoting the release of pro-inflammatory cytokines (e.g., TNF-α, IL-6, and IL-1β), thereby triggering persistent inflammation, chronic airway injury, and pulmonary remodeling, ultimately driving COPD pathogenesis [11,12,13]. Clinical evidence shows elevated serum CRP, leukocyte counts, and IL-6/8 levels in COPD patients [14]. Notably, during acute exacerbations (AECOPD), 8-OHdG levels are significantly higher in smokers and correlate with disease severity and inflammatory markers compared to healthy controls or stable COPD patients [15]. Current COPD management relies on pharmacotherapy (e.g., bronchodilators, corticosteroids, and antioxidants) and non-pharmacological interventions (e.g., pulmonary rehabilitation) [16,17,18]. While these approaches alleviate symptoms, they fail to halt or reverse disease progression and may pose safety concerns due to long-term side effects [18,19].

Given the aforementioned therapeutic challenges, modern functional foods offer innovative solutions for COPD management. The functional role of contemporary foods has evolved beyond merely providing basic nutrition to encompass disease prevention and therapeutic benefits [20,21,22]. Epidemiological evidence suggests that diets rich in antioxidants may improve lung function in patients with chronic respiratory diseases [23,24,25]. Studies have demonstrated that numerous food-derived bioactive compounds—such as resveratrol, curcumin, and quercetin—can enhance pulmonary function by modulating oxidative stress and inflammation, while their natural food matrix ensures long-term safety for regular consumption [26,27,28]. In this context, marine bioactive peptides exhibit unique advantages. On one hand, as previously discussed, many marine-derived peptides possess confirmed antioxidant, anti-inflammatory, and anti-apoptotic properties, enabling them to target key pathological mechanisms of COPD. On the other hand, advancements in food processing technologies—such as Maillard reaction modification and nanoencapsulation—have effectively addressed challenges related to bitterness and stability [29]. For instance, nanoencapsulation of fish-derived antioxidant peptide F5 within chitosan–lipid nanoparticles enhances its thermal stability while preserving free radical scavenging capacity [30]. Similarly, Maillard reaction-modified sea cucumber hydrolysate (CFH) demonstrates significantly improved palatability, facilitating its incorporation into functional beverages or dietary supplements [31]. These findings highlight the potential of modern functional foods to provide COPD patients with safe, feasible, and sustainable options for symptom alleviation and quality-of-life improvement [32].

Building upon the demonstrated potential of marine bioactive peptides in functional food applications, we investigated eight bioactive peptides previously isolated and identified from skipjack tuna surimi—DVGRG (S1), LEPH (S2), AEHNH (S3), GHHAA (S4), PHPR (S5), GRVPR (S6), SVTEV (S7), and VRDQY (S8). Using these tuna-derived peptides (S1–S8) as our research subjects, we examined their protective effects against cigarette smoke extract (CSE)-induced chronic obstructive pulmonary disease (COPD) in cellular models [33]. Furthermore, we elucidated the mechanisms by which these peptides alleviate oxidative stress, suppress inflammatory responses, and modulate apoptosis-related pathways in COPD models. This study not only provides novel mechanistic insights into the therapeutic potential of marine bioactive peptides but also establishes a foundation for developing functional foods or novel therapeutic agents for the prevention and treatment of chronic obstructive pulmonary disease.

## 2. Results

### 2.1. Screening CSE Concentrations and Evaluating Cytoprotective Effects of Tuna Peptides

**Figure 1:**
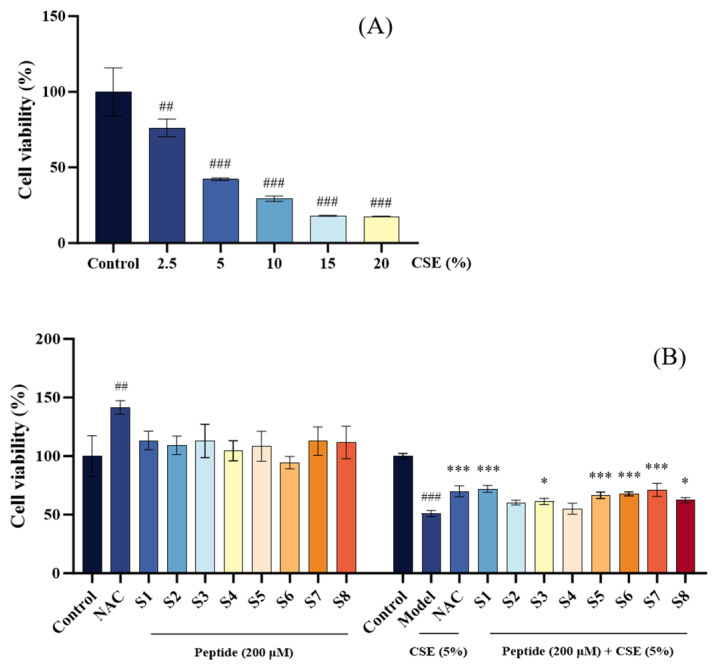
Cytotoxicity and protective effects of tuna peptides on MLE-12 cells. (**A**) Screening of cigarette smoke particle concentration. (**B**) Cytotoxicity assessment of tuna peptides on MLE-12 cells and protective effects on CSE-damaged MLE-12 cells. Control refers to the unprocessed normal cells. Model refers to CSE-damaged cells without peptides. N-Acetylcysteine (NAC) at 300 μM was served as the positive control. ^###^ *p* < 0.001 and ^##^ *p* < 0.001 compared with Control; *** *p* < 0.001 and * *p* < 0.05 compared with Model.

Initial screening of CSE concentrations revealed a dose-dependent decrease in MLE-12 cell viability, with a 5% CSE solution reducing viability to 50.98 ± 1.61% (*p* < 0.001 vs. control), establishing this concentration as optimal for inducing COPD-like cellular damage in subsequent experiments (Figure 1A).

To assess the safety of tuna peptides, cells were treated with 200 µM of peptide S1–8, which maintained viability above 90%, comparable to the blank control (100.00 ± 18.18%) and non-toxic relative to the positive control (134.31 ± 10.75%) (Figure 1B). In the CSE-induced injury model (cell viability: 50.89 ± 2.68% vs. control), pretreatment with tuna peptides S1, S5, S6, and S7 significantly restored viability, with S6 and S7 exhibiting efficacy comparable to the positive drug (*p* < 0.001) and outperforming peptides S2–S4 and S8 (Figure 1B). These findings identify S1, S5, S6, and S7 as promising candidates for further mechanistic studies.

### 2.2. Assessment of Oxidative Stress in CSE-Damaged MLE-12 Cells Treated with Tuna Peptides

#### 2.2.1. Measurement of ROS Levels

**Figure 2:**
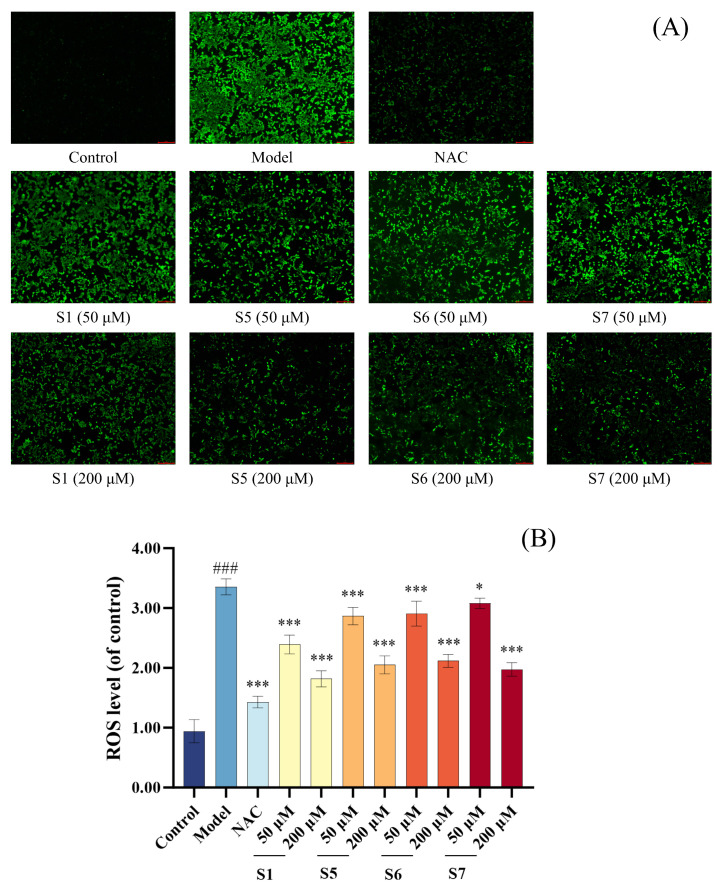
Quantitative analysis of the effect of different concentrations of tuna peptides on the intracellular ROS content in CSE-damaged MLE-12 cells (**A**,**B**). ^###^ *p* < 0.001 compared with Control; *** *p* < 0.001 and * *p* < 0.05 compared with Model.

Figure 2A,B illustrate the effects of tuna peptides S1, S5, S6, and S7 on ROS levels in CSE-induced MLE-12 cells. The DCFH-DA staining revealed a notable increase in green fluorescence intensity and distribution area in the model group compared to the control group, indicating elevated intracellular ROS levels. As the concentrations of tuna peptides S1, S5, S6, and S7 increased, both fluorescence intensity and ROS-positive areas exhibited a downward trend, suggesting a dose-dependent ROS-scavenging effect. Quantitative analysis further confirmed that the intracellular ROS levels in the model group were significantly higher than those in the blank control group (*p* < 0.001). However, pretreatment with S1, S5, S6, and S7 significantly reduced ROS levels in a concentration-dependent manner. These results demonstrate that tuna peptides S1, S5, S6, and S7 effectively mitigate oxidative stress by reducing intracellular ROS accumulation in CSE-exposed MLE-12 cells.

#### 2.2.2. Evaluation of Antioxidant Indicators

**Figure 3:**
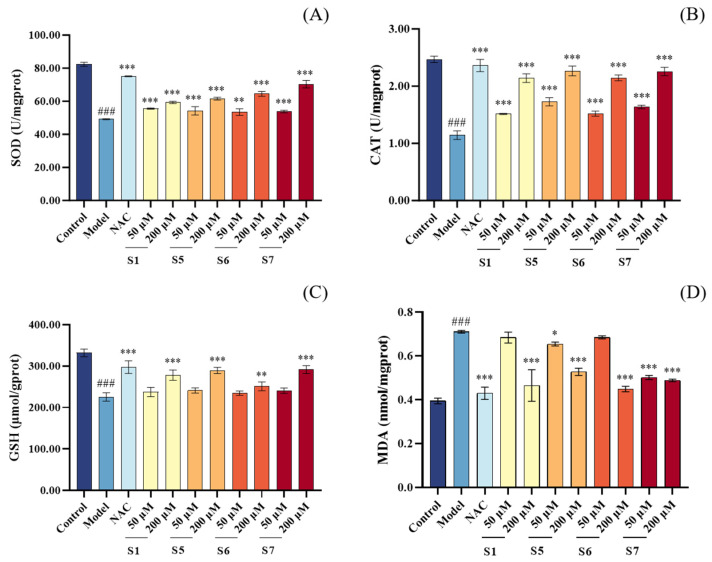
The effect of different concentrations of tuna peptides on the levels of SOD (**A**), CAT (**B**), GSH (**C**), and MDA (**D**) in CSE-damaged MLE-12 cells. ^###^ *p* < 0.001 compared with Control; *** *p* < 0.001, ** *p* < 0.01, and * *p* < 0.05 compared with Model.

Compared to the control group, the model group exhibited significantly decreased levels of CAT, SOD, and GSH, along with a marked increase in MDA levels (*p* < 0.001), as shown in Figure 3. In comparison to the model group, all tuna peptide treatment groups at a concentration of 200 μM demonstrated enhanced cellular antioxidant capacity, characterized by increased CAT and SOD activities, elevated GSH levels, and reduced MDA levels (*p* < 0.001). Notably, the 200 μM concentration of tuna peptides showed a more pronounced ameliorative effect on CSE-induced oxidative stress in MLE-12 cells than the 50 μM concentration. These results indicate that tuna peptides effectively alleviate CSE induced oxidative stress by enhancing antioxidant enzyme activity and reducing lipid peroxidation.

### 2.3. Assessment of Pro-Inflammatory Cytokine Production in CSE-Damaged MLE-12 Cells Treated with Tuna Peptides

**Figure 4:**
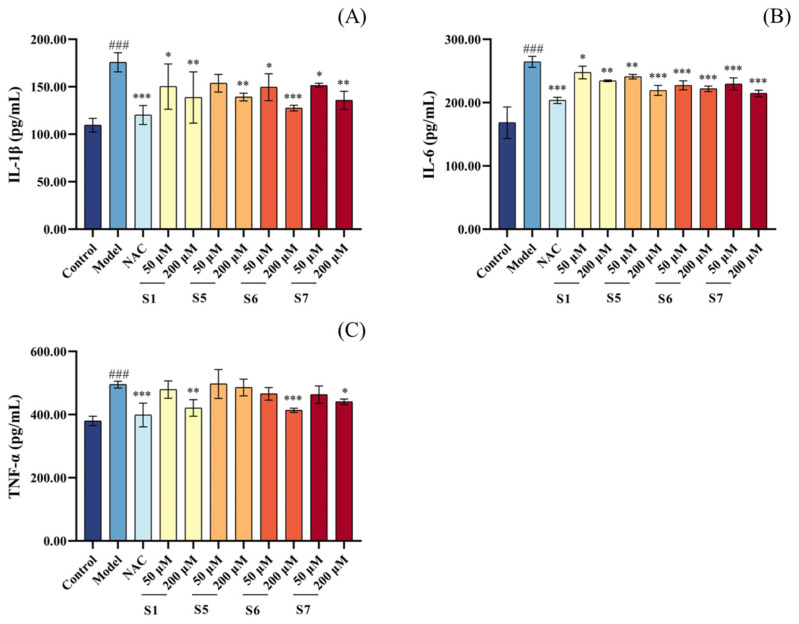
Different concentrations of tuna peptides affect the intracellular IL-1β (**A**), IL-6 (**B**), and TNF-α (**C**) contents in CSE-damaged MLE-12 cells. ^###^ *p* < 0.001 compared with Control; *** *p* < 0.001, ** *p* < 0.01, and * *p* < 0.05 compared with Model.

As shown in Figure 4, the levels of IL-1β, IL-6, and TNF-α in the model group were significantly higher than those in the blank control group (*p* < 0.001), confirming the upregulation of pro-inflammatory cytokines induced by CSE exposure. As the concentrations of peptides S1, S5, S6, and S7 increased from 50 µM to 200 µM, the intracellular levels of IL-1β, IL-6, and TNF-α showed a decreasing trend. At a concentration of 200 μM, the tuna peptide treatment groups (S1, S6, and S7) showed a significant reduction in these inflammatory markers compared to the model group. Overall, tuna peptide S6 demonstrated the strongest anti-inflammatory effect, suggesting its potential as a promising candidate peptide for mitigating inflammatory responses. These findings indicate that pre-treatment with tuna peptides S1, S5, S6, and S7 significantly reduces the intracellular levels of inflammatory cytokines (IL-1 β, IL-6, and TNF-α), thereby effectively reducing CSE-induced inflammation levels in MLE-12 cells.

### 2.4. Assessment of Apoptosis in CSE-Damaged MLE-12 Cells Treated with Tuna Peptides

#### 2.4.1. Quantification of Apoptosis by Flow Cytometry

**Figure 5:**
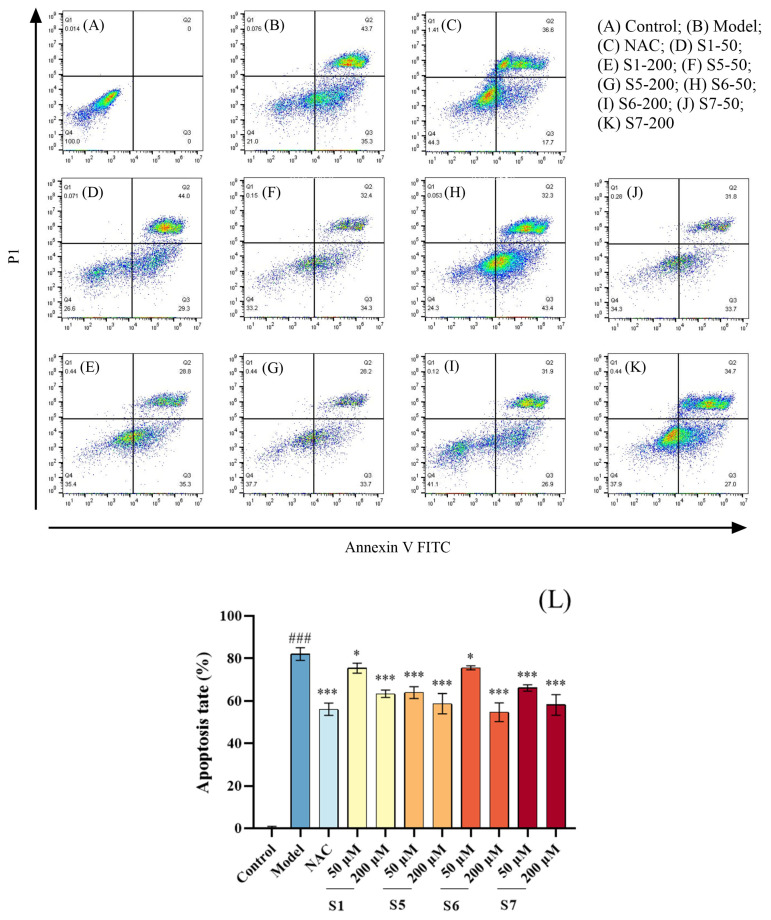
Effect of tuna peptides on apoptosis of MLE-12 cells after CSE treatment (**A**–**L**). ^###^ *p* < 0.001 compared with Control; *** *p* < 0.001, * *p* < 0.05 compared with Model.

As illustrated in Figure 5, the proportion of apoptotic cells in the model group (C) was significantly higher compared to the control group (A) (*p* < 0.001), indicating that a substantial number of MLE-12 cells were damaged by CSE and entered an apoptotic state. With increasing concentrations of tuna peptides S1, S5, S6, and S7, the proportion of apoptotic cells decreased. At a concentration of 200 µM, the apoptosis rate in the peptide-treated groups was significantly lower than that in the model group (*p* < 0.001). Among these, the 200 µM concentration of peptide S6 exhibited a stronger inhibitory effect on CSE-induced apoptosis in MLE-12 cells. In summary, pretreatment with tuna peptides S1, S5, S6, and S7 significantly reduced the number of CSE induced apoptosis in MLE-12 cells.

#### 2.4.2. Measurement of Mitochondrial Membrane Potential (MMP)

**Figure 6:**
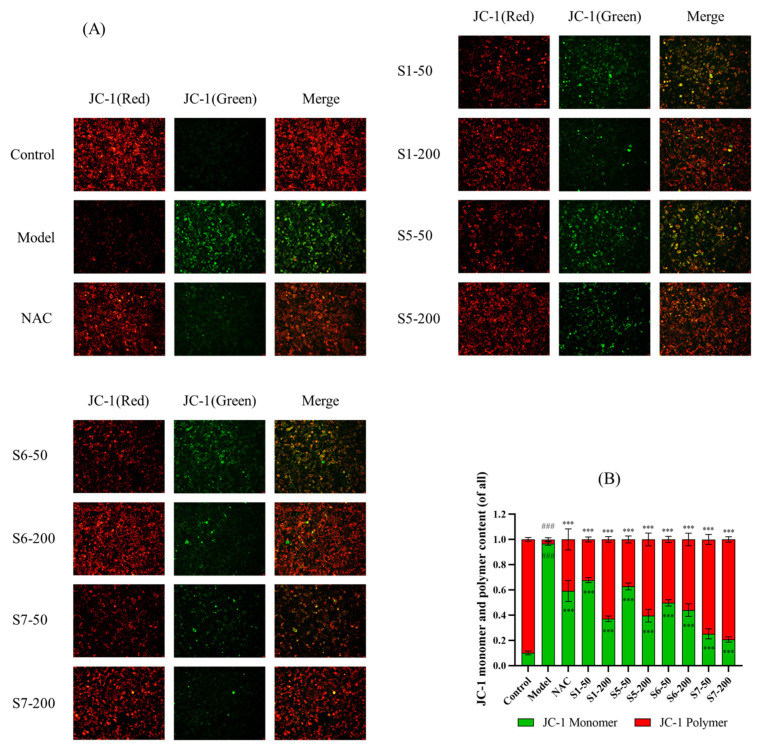
The effects of tuna peptides on the mitochondrial membrane potential (MMP) in CSE-treated MLE-12 cells (**A**,**B**). ^###^ *p* < 0.001 compared with Control; *** *p* < 0.001 compared with Model.

The MMP is a critical indicator of early apoptosis. Under normal conditions, high MMP promotes JC-1 aggregation, resulting in red fluorescence, whereas a decrease in MMP causes JC-1 dissociation, leading to green fluorescence emissions. As shown in Figure 6, the model group exhibited a significant increase in green fluorescence and a marked decrease in red fluorescence compared to the normal control group (*p* < 0.001), indicating that CSE treatment led to mitochondrial dysfunction and apoptosis in MLE-12 cells. However, treatment with tuna peptides S1, S5, S6, and S7, resulted in a significant reduction in green fluorescence and a notable increase in red fluorescence (*p* < 0.001), suggesting that these peptides effectively restored MMP and inhibited CSE-induced apoptosis. These findings indicate that tuna peptides S1, S5, S6, and S7 exert a protective effect against CSE-induced mitochondrial dysfunction, thereby reducing apoptosis in MLE-12 cells.

### 2.5. Assessment of Cell Migration in CSE-Damaged MLE-12 Cells Treated with Tuna Peptides

**Figure 7:**
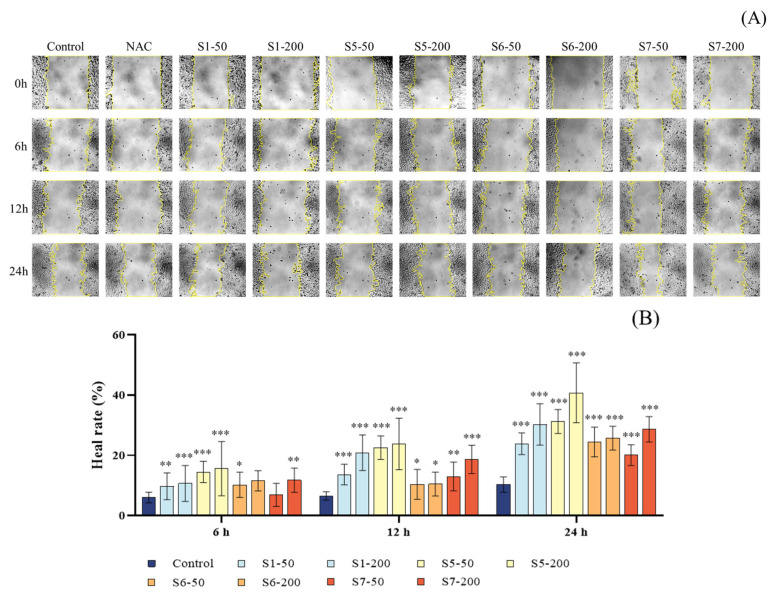
Effect of different peptide concentrations on the healing of scratches in CSE-damaged MLE-12 cells. (**A**) Wound healing process. (**B**) Quantitative analysis of wound healing rate. Yellow color in the figure A represents the degree of wound healing. *** *p* < 0.001, ** *p* < 0.01, and * *p* < 0.05 compared with Control.

The scratch assay was used to evaluate the effect of CSE on the migration ability of MLE-12 cells. As shown in Figure 7A, the microscopic images of the control group and the groups treated with tuna peptides S1, S5, S6, and S7 demonstrated differences in cell migration. As shown in Figure 7B, compared to the control group, the migration rates of MLE-12 cells treated with different concentrations of tuna peptides S1, S5, S6, and S7 at 0 h, 6 h, 12 h, and 24 h were significantly increased, with statistically significant differences (*p* < 0.05). These results indicate that tuna peptides S1, S5, S6, and S7 significantly promote the migration of MLE-12 cells and accelerate wound closure in a concentration-dependent manner.

### 2.6. Mechanistic Studies

#### 2.6.1. Antioxidant Mechanisms

##### mRNA Expression of Keap1 and Nrf2

**Figure 8:**
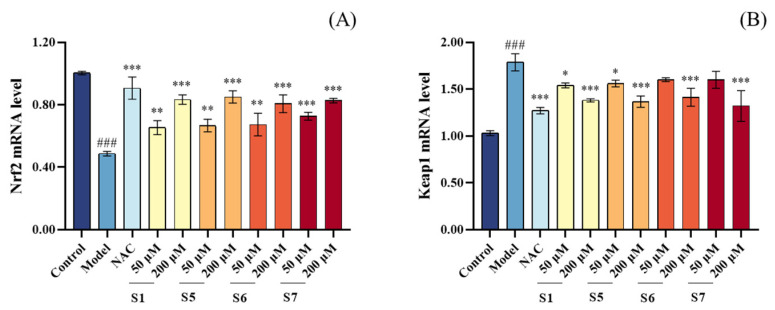
Effect of different concentrations of peptide on the mRNA content of Nrf2 (**A**) and Keap1 (**B**) in MLE-12 cells. ^###^ *p* < 0.001 compared with Control; *** *p* < 0.001, ** *p* < 0.01, * *p* < 0.05 compared with Model.

The Keap1/Nrf2 pathway is one of the key pathways involved in cellular antioxidant responses. As shown in Figure 8, the model group exhibited a significant decrease in Nrf2 mRNA expression and a notable increase in Keap1 mRNA expression compared to the control group, indicating that CSE exposure disrupted the Keap1/Nrf2 axis. In contrast, pretreatment with 200 μM tuna peptides S1, S5, S6, and S7 significantly increased Nrf2 mRNA expression and decreased Keap1 mRNA expression in MLE-12 cells compared to the model group. This suggests that tuna peptides effectively activate the Keap1/Nrf2 signaling pathway. Furthermore, the activation of this pathway enhanced the expression and activity of downstream antioxidant enzymes, including SOD, CAT, and GSH-Px, which aligns with the increased antioxidant enzyme activity observed in previous experiments. These findings demonstrate that tuna peptides S1, S5, S6, and S7 bolster cellular antioxidant defenses, mitigate oxidative stress, and exert significant cytoprotective effects against CSE-induced damage.

##### Nrf2 Nuclear Translocation by Immunofluorescence

**Figure 9:**
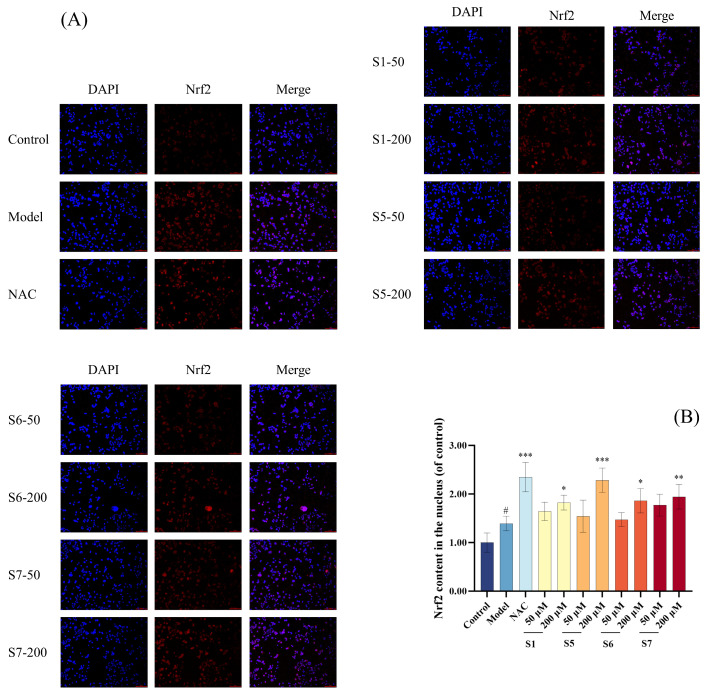
The effect of different concentrations of peptides on the Nrf2 content in the nucleus of MLE-12 cells (**A**,**B**). ^#^ *p* < 0.001 compared with Control; *** *p* < 0.001, ** *p* < 0.01, * *p* < 0.05 compared with Model.

As shown in Figure 9, the nuclear Nrf2 content in the model group was significantly higher compared to the blank group (*p* < 0.05), indicating that MLE-12 cells underwent oxidative stress, and Nrf2 dissociated from Keap1 and entered the nucleus. At a concentration of 200 μM, the nuclear Nrf2 levels in the S1 and S6 peptide-treated groups showed significant higher compared to the model group (*p* < 0.05). In contrast, the S5 peptide-treated group exhibited an extremely significant increase (*p* < 0.001), while the S7 peptide-treated group demonstrated a highly significant increase (*p* < 0.01) compared to the model group. These results indicate that tuna peptides S1, S5, S6, and S7 can enhance the transcriptional activation of Nrf2 by promoting its nuclear translocation, which in turn mitigates CSE-induced oxidative damage.

##### Molecular Docking of Tuna Peptides with Keap1

**Figure 10:**
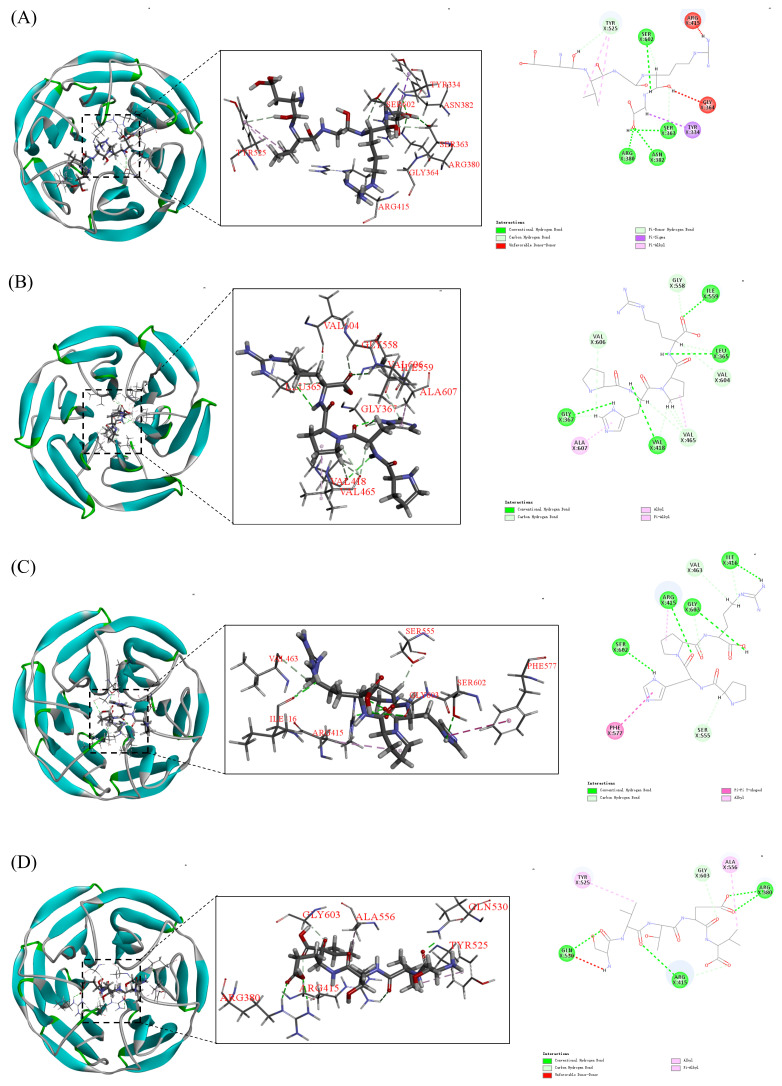
Molecular docking model of peptide S1, S5, S6, and S7 with Kelch region of Keap1 protein (**A**–**D**).

Molecular docking was employed to investigate the binding patterns and interactions between tuna peptides S1, S5, S6, and S7 and the Kelch domain of Keap1 (Figure 10).

The results, as shown in Figure 10 and Table 1, indicated that peptide S6 exhibited the strongest binding energy (−7.48 kcal/mol), followed by peptides S5, S1, and S7. Specifically, Tuna peptide S6 (GRVPR) formed six hydrogen bonds with the Kelch region, mainly involving Arg415, Gly603, Val463, Ile416, Ser555, and Ser602. Additionally, it established hydrophobic interactions with Phe577, with Arg415 located in the active pocket P1, Gly603, Ser555, and Ser602 in the active pocket P3, and Phe577 in the active pocket P5. These interactions indicate a strong affinity of S6 for Keap1. The other peptides (S1, S5, and S7) also showed a stronger binding ability to Keap1 protein, and the specific binding energies, number of hydrogen bonds, and regions of hydrophobic interaction are detailed in Table 1. It is noteworthy that tuna peptide S5 formed hydrogen bonds with Gly558, Ile559, Leu365, Val604, Val465, Gly367, Val606, and Val418 in the Kelch region of Keap1 and hydrophobic interaction force with Ala607. However, none of these binding sites were located in the active pockets, suggesting that peptide S5 may exert a non-competitive inhibition mechanism by preventing the binding of Keap1 to Nrf2.

#### 2.6.2. Anti-Inflammatory Mechanisms

**Figure 11:**
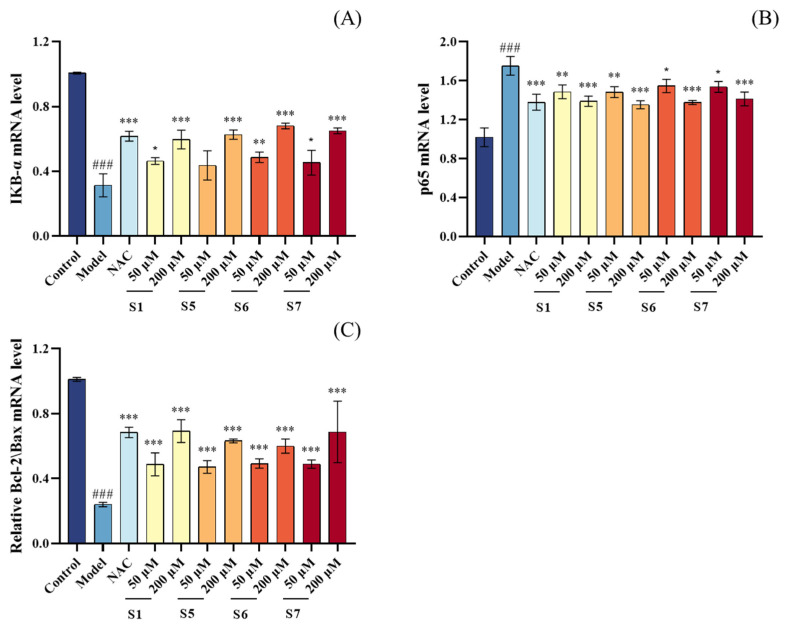
Effect of different concentrations of peptide on the mRNA content of p65 (**A**), IκBα (**B**) and Bcl-2/Bax mRNA (**C**) in MLE-12 cells. ^###^ *p* < 0.001 compared with Control; *** *p* < 0.001, ** *p* < 0.01, * *p* < 0.05 compared with Model.

As shown in Figure 11A,B, compared to the control group, the model group exhibited a significant increase in p65 mRNA expression and a significant decrease in IκBα mRNA expression (*p* < 0.001), suggesting that CSE-induced oxidative stress may activate the NF-κB signaling pathway. However, treatment with 200 µM tuna peptides S1, S5, S6, and S7 significantly reduced p65 mRNA expression and increased IκBα mRNA expression (*p* < 0.001). This result may be attributed to the effective suppression of intracellular oxidative stress by tuna peptides, thereby reducing the downstream activation of the NF-κB signaling pathway and preventing the overexpression of p65. In conclusion, tuna peptides alleviate CSE-induced inflammation by inhibiting the NF-κB signaling pathway, thereby exerting anti-inflammatory effects.

#### 2.6.3. Anti-Apoptotic Mechanisms

As shown in Figure 11C, compared to the control group, the model group exhibited a significant decrease in the Bcl-2/Bax mRNA expression ratio, with highly significant differences (*p* < 0.001). In contrast, pretreatment with 200 μM tuna peptides S1, S5, S6, and S7 significantly increased the Bcl-2/Bax mRNA expression ratio compared to the model group, with highly significant differences (*p* < 0.001). These results indicate that tuna peptides S1, S5, S6, and S7 reduce the likelihood of cell apoptosis by promoting the expression of the anti-apoptotic factor Bcl-2, inhibiting the expression of the pro-apoptotic factor Bax, and modulating the Bcl-2/Bax ratio. This finding is consistent with previous flow cytometry results, which demonstrated that tuna peptides S1, S5, S6, and S7 inhibit cell apoptosis.

## 3. Discussion

Smoking is the primary cause of COPD in high-income countries, accounting for approximately 70% of cases. The fine particles in cigarette smoke deposit in the smaller peripheral airways and alveolar sacs, leading to chronic inflammation, infection, oxidative stress, and damage to the airways and lung gas exchange regions. Oxidative stress plays a central role in COPD pathogenesis, occurring when ROS levels exceed the capacity of endogenous antioxidant defenses [34]. In addition to direct ROS exposure from cigarette smoke, activated inflammatory cells in the body also contribute to ROS production [35]. Notably, even after smoking cessation, persistent ROS production by activated inflammatory cells can sustain high oxidative stress levels, potentially driving disease progression [36]. In line with this concept, our study demonstrates that tuna peptides S1, S5, S6, and S7 enhance antioxidant capacity by increasing SOD, CAT, and GSH-Px activities while reducing MDA and ROS levels. These findings align with previous research on marine-derived peptides, such as the peptide PGY, which upregulates CAT and SOD in HepG2 cells, and other antioxidant peptides that inhibit lipid peroxidation [37,38].

**Figure 12:**
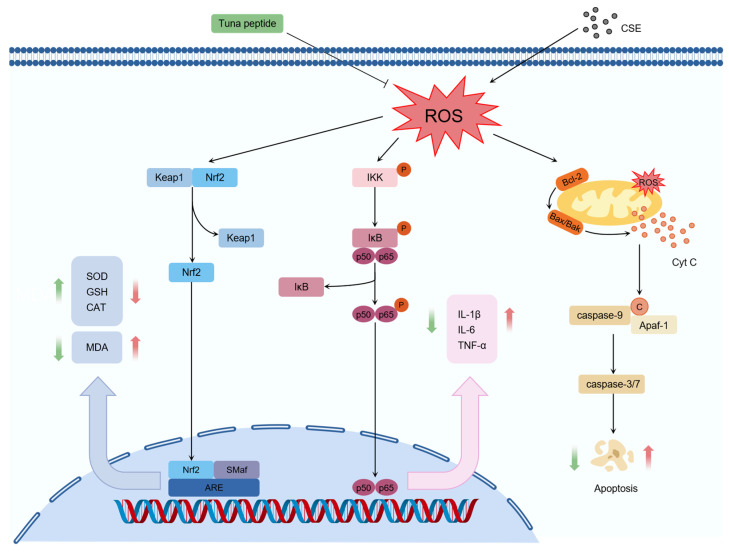
Tuna peptides protect MLE-12 cells from the effects of CSE by regulating Keap1/Nrf2, NF-κB signaling pathways, and regulating Bcl-2/Bax expression. In the figure, black arrows indicate promotion, black blunt-ended arrows indicate inhibition, red arrows represent changes in these substances induced by peptides, and green arrows represent changes in these substances induced by CSE.

Beyond their antioxidant effects, we further explored the underlying mechanisms of these peptides. Mechanistic studies revealed that S1, S5, S6, and S7 exert their antioxidant effects by regulating the Keap1/Nrf2 signaling pathway (Figure 12). Beata Kosmider et al. showed that Nrf2 activation protects human alveolar epithelial cells against CSE-induced oxidative stress, suggesting that Nrf2-targeting antioxidants could restore antioxidant enzyme activities, prevent lung injury, and potentially attenuate emphysema development [39]. Molecular docking analysis provided additional insights, revealing that peptides S1, S5, and S6 can bind strongly to the Keap1 Kelch region, while S1, S6, and S7 can competitively inhibit Nrf2 binding by occupying the Keap1-Nrf2 interaction site. Interestingly, S5 appears to act via a non-competitive mechanism, as its binding sites on Keap1 (Leu365, Val465, Gly367, Val606, and Val418) overlap with those of the known antioxidant peptide TCP3 [40]. These results corroborate earlier studies suggesting that disrupting Keap1-Nrf2 interactions can enhance antioxidant defenses [41,42].

In addition to redox modulation, the tested peptides suppressed the production of pro-inflammatory cytokines, which are typically upregulated in response to cigarette smoke-induced oxidative damage. In this study, we found that S1, S5, S6, and S7 significantly reduced pro-inflammatory cytokines expression in CSE-induced MLE-12 cells by inhibiting NF-κB pathway activation. This effect was mediated by down-regulating NF-κB signaling molecule p65 mRNA and up-regulating IκBα mRNA expression (Figure 12). This mechanism mirrors findings from other marine-derived peptides, such as C-phycocyanin peptides and marine collagen peptides, which also modulate NF-κB signaling to reduce inflammation [43,44].

Apoptosis, particularly via the mitochondrial pathway, further contributes to COPD progression. Imbalances in pro-apoptotic (e.g., Bax) and anti-apoptotic (e.g., Bcl-2) proteins disrupt mitochondrial membrane potential, triggering cytochrome C release and caspase-3 activation [13,45]. Our results indicate that S1, S5, S6, and S7 can restore mitochondrial membrane potential and inhibit apoptosis in CSE-induced MLE-12 cells by upregulating the Bcl-2/Bax mRNA expression ratio (Figure 12). Similar protective effects have been reported for other marine peptides, such as PIISVYWK and FSVVPSPK from blue mussels, which regulate apoptosis-related proteins [46].

Pulmonary epithelial cells constitute the first line of defense against inhaled pathogens and harmful particles. Chronic exposure to cigarette smoke triggers oxidative stress, inflammation, apoptosis, and cellular dysfunction, all of which contribute to COPD pathogenesis [47]. Impaired epithelial regeneration exacerbates airway remodeling, inflammation, and fibrosis, further driving disease progression [48]. We found that S1, S5, S6, and S7 promote the migration of CSE-damaged MLE-12 cells, suggesting a potential role in epithelial regeneration. We hypothesize that this effect stems from their ability to reduce oxidative stress and inflammation, creating a favorable microenvironment for cell repair. This observation is supported by studies on other marine bioactive compounds, such as pearl oyster mucin protein (PMP), which enhances fibroblast migration and wound healing [49].

In summary, our study demonstrates that tuna peptides S1, S5, S6, and S7 exhibit potent antioxidant, anti-inflammatory, and anti-apoptotic activities in CSE-induced MLE-12 cells, primarily through the regulation of the Keap1/Nrf2 and NF-κB signaling pathways. These bioactivities suggest that marine peptides may serve as valuable candidates for incorporation into functional foods designed to support respiratory resilience in smokers or individuals exposed to polluted environments. Future research should explore the applicability of these peptides in in vivo models of smoke-induced lung injury, assess their bioavailability after oral administration, and evaluate their sensory properties and stability in functional food matrices. Ultimately, such marine-derived peptides hold promise as natural dietary agents capable of modulating oxidative stress and maintaining epithelial health under environmental challenges.

## 4. Materials and Methods

### 4.1. Materials and Reagents

The tuna peptide S1–S8 (DVGRG, LEPH, AEHNH, GHHAA, PHPR, GRVPR, SVTEV, VRDQY, >95% purity) was synthesized by Shanghai Apeptide Co., Ltd. (Shanghai, China). Mouse lung epithelial cells (MLE-12) were obtained from Wuhan Procell Life Science & Technology Co., Ltd. (Wuhan, China). Additional reagents and kits were obtained from various suppliers: BCA Protein Assay Kits and RNA easy™ Animal RNA Extraction Kit from Beyotime Biotechnology (Shanghai, China); 2X Universal SYBR Green Fast qPCR Mix and ABScript III RT Master Mix for qPCR with gDNA Remover from ABclonal Biotechnology Co., Ltd. (Wuhan, China). The Annexin V-FITC/PI Staining Kit was purchased from Becton Dickinson (Franklin Lakes, NJ, USA), while the Mitochondrial Membrane Potential Assay Kit (JC-1) was obtained from Solarbio Biotechnology Co., Ltd. (Beijing, China).

### 4.2. Cigarette Smoke Extract (CSE) Collection

The CSE was prepared using commercially available Taishan brand cigarettes (China Tobacco Shandong Industrial Co., Ltd., Jinan, China), with the following specifications: 8 mg tar/cigarette, 0.8 mg nicotine/cigarette, and 10 mg carbon monoxide/cigarette. A 75 mL Duran glass bottle containing 20 mL of MLE-12 cell culture medium was used as the smoke absorption solution. One end of the bottle was connected to a cigarette, while the other end was attached to a 50 mL syringe to simulate inhalation. After lighting the cigarette, the smoke was drawn evenly 15 times until the cigarette was fully burned, while the bottle was gently shaken to facilitate complete dissolution of the smoke components. The collected solution was then filtered to remove particulates, and its pH was adjusted to 7.2–7.4. The absorbance was measured at 320–540 nm to ensure an OD value between 0.9 and 1.2 to confirm the consistency of the 100% CSE stock solution. The stock solution is aliquoted and stored at −80 °C. Before experiments, the CSE stock solution was diluted to the required concentrations, and all prepared CSE solutions were used within 30 m to maintain stability. Any remaining unused CSE was discarded to prevent degradation.

### 4.3. MLE-12 Cell Culture and the Establishment of COPD Model

MLE-12 cells were cultured in a humidified incubator at 37 °C with 5% CO_2_. The culture medium consisted of DMEM/F-12 supplemented with 10% fetal bovine serum (FBS), 100 U/mL penicillin, and 100 μg/mL streptomycin. MLE-12 cells were seeded in 96-well plates and cultured for 24 h. After incubation, the culture medium in the control group was replaced with fresh medium, while the experimental groups were treated with CSE solutions at concentrations of 2.5%, 5%, 10%, 15%, and 20%, respectively. Following 24 h of exposure, the treatment medium was replaced with fresh medium, and the cells were further cultured for an additional 24 h. Cell viability was then assessed using the CCK-8 assay. This modeling method is the same as that of Dai et al. [50].

Cell viability (%) = OD_Experimental group_/OD_Blank group_ × 100% (1)

### 4.4. Cytotoxicity Assessment of Tuna Peptide

Cells at 80% confluence were digested, centrifuged, and suspended. The cell density was adjusted to 1.2 × 10^4^ cells/well, and 180 μL of cell suspension was seeded into each well of a 96-well plate. Following 24 h of incubation, treatments were administered: 20 μL of PBS for the blank control group, 300 μM NAC for the positive control group, and 200 μM tuna peptide for the experimental group. After 24 h of treatment, cell viability was assessed by incubating with 10% CCK-8 solution for 1 h, followed by measurement of optical density at 450 nm [51].

### 4.5. Screening of Protective Effects of Tuna Peptides on CSE-Damaged MLE-12 Cells

The cell culture and seeding methods followed the procedure described in Section 4.3. The experimental grouping and processing steps were as follows: After the cells adhered and grew for 24 h, the blank control group and the model group were treated with 180 μL of cell culture medium and 20 μL of PBS solution per well. The positive control group received 300 mM NAC solution, while the treatment groups were administered 200 μM of individual tuna peptide solutions (S1–S8). After 24 h of pre-treatment, CSE was added to the wells except the blank well, and the cells were incubated for another 24 h to induce damage. Cell viability was then assessed using the Cell Counting Kit-8 (CCK-8) assay, and the survival rate of each group was calculated accordingly.

### 4.6. Antioxidant Activity of Tuna Peptides in CSE-Damaged MLE-12 Cells

#### 4.6.1. Measurement of Intracellular Reactive Oxygen Species (ROS)

To evaluate the effects of CSE and tuna peptide on oxidative stress and inflammation, intracellular ROS levels were measured using the DCFH-DA fluorescent probe [40]. Briefly, cells were washed with pre-cooled PBS and incubated with 10 μM DCFH-DA in the dark at 37 °C for 90 m. After incubation, cells were washed with PBS to remove excess probe, and fluorescence images were captured using a fluorescence microscope. (Nikon Corporation, Tokyo, Japan). ROS levels were quantified based on fluorescence intensity.

#### 4.6.2. Analysis of Oxidative Stress Markers

The levels of oxidative stress markers were determined in the experiment described by Hu et al. [52]. Oxidative stress markers, including malondialdehyde (MDA), superoxide dismutase (SOD), catalase (CAT), and reduced glutathione (GSH), were measured using commercially available assay kits from Nanjing Jiancheng Bioengineering Institute (Nanjing, China). All assays were performed in accordance with the manufacturers’ protocols.

### 4.7. Anti-Inflammatory Effects of Tuna Peptides in CSE-Damaged MLE-12 Cells

In the experiment described by Zheng et al., the levels of inflammatory factors in the cells were measured [53]. Inflammatory factors, including tumor necrosis factor-alpha (TNF-α), interleukin-1 beta (IL-1β), and interleukin-6 (IL-6), were measured using ELISA kits from Shanghai Enzyme Linked Biotechnology Co., Ltd. (Shanghai, China). All assays were performed in accordance with the manufacturers’ protocols.

### 4.8. Anti-Apoptotic Effects of Tuna Peptides in CSE-Damaged MLE-12 Cells

#### 4.8.1. Measurement of Mitochondrial Membrane Potential (MMP)

MMP was determined using previous method [54]. The MMP of MLE-12 cells was assessed using the Mitochondrial Membrane Potential Assay Kit (JC-1) (Solarbio, Beijing, China). Briefly, the JC-1 staining solution (200×) was diluted with ddH_2_O and JC-1 buffer (5×) to prepare the JC-1 working solution, following the manufacturer’s instructions. Cells were stained with the JC-1 working solution and incubated at 37 °C for 15–20 min. After incubation, cells were washed with PBS to remove excess staining solution, and fluorescence images were captured and recorded using a fluorescence microscope.

#### 4.8.2. Annexin V-FITC/PI Staining for Apoptosis Detection

In the experiment described by Pan et al., the level of cell apoptosis was measured [55]. After 24 h of drug treatment, cell apoptosis was assessed using the Annexin V-FITC/PI staining kit according to the manufacturer’s protocol. Briefly, binding buffer was diluted 10-fold with ddH_2_O to prepare 1 × Binding buffer. Cells were collected, washed with PBS, and resuspended at a density of 3 × 10^6^ cells/mL, followed by centrifugation at 2000 rpm for 5–10 min. The cell pellet was resuspended in 300 µL of 1 × Binding Buffer and stained with 5 µL Annexin V-FITC in the dark for 15 min. Prior to analysis, 5 µL propidium iodide (PI) was added, and the volume was adjusted to 500 µL with 1 × Binding Buffer. Cell viability and apoptosis were analyzed using a FACScan flow cytometer. (BD Biosciences, San Jose, CA, USA).

### 4.9. Scratch Assay

The cell scratch assay described by Xu et al. [56]. A scratch assay was conducted to assess the effects of tuna peptides on MLE-12 cell migration. Prior to cell seeding, a marker and ruler were used to draw evenly spaced horizontal reference lines (0.5–1 cm apart) on the back of a 6-well plate, ensuring that at least five lines intersected each well.

Cells were cultured and grouped as described in Section 4.5, and each well was treated with corresponding concentrations of NAC and peptides S1, S5, S6, and S7 for 24 h. The following day, a sterile pipette tip was used to create a vertical scratch perpendicular to the pre-drawn horizontal reference lines, ensuring a consistent scratch width by holding the pipette vertically. Detached cells were removed by washing three times with PBS, and serum-free medium was added to eliminate the influence of cell proliferation. The plate was incubated at 37 °C with 5% CO_2_, and images were captured at 0, 6, 12, and 24 h to evaluate the rate of wound closure.

### 4.10. Immunofluorescence

According to the description by Liu et al., the content of Nrf2 in the cell nucleus was detected using the immunofluorescence method [57]. The cells were cultured and grouped as described in Section 4.5. After the cell culture was completed, the culture medium was discarded and 4% paraformaldehyde was added to fix the cells for 30 min. After the cells were fixed, a blocking solution (containing 5% goat serum and 0.3% Triton) was added and the cells were incubated on a shaker for 1 h. Then, an Nrf2 antibody (1:200, containing 1% BSA and 0.3% Triton) was added and incubated at 4 °C overnight. After washing with PBS, a fluorescent secondary antibody (1:500) was added and incubated on a shaker at room temperature for 1 h. Finally, DAPI dye was used to stain the nuclei for 10 min and observed under a fluorescence microscope. The fluorescence signals within the cells in different fluorescence channels were recorded.

### 4.11. Real-Time PCR

The determination method of RT-PCR was described by Wei et al. [58]. To elucidate the protective mechanisms of tuna peptide against CSE-induced injury in MLE-12 cells, the mRNA expression levels of genes associated with antioxidant (Keap1, Nrf2), anti-inflammatory (NfκB-P65, IκBα), and anti-apoptotic (Bcl-2, Bax) pathways were analyzed using RT-PCR. Total RNA was extracted using the RNA easy™ Animal RNA Extraction Kit (Beyotime Biotechnology, Shanghai, China), and its concentration was measured using a spectrophotometer. Subsequently, RNA was reverse transcribed into cDNA using the ABScript III RT Master Mix (ABclonal Biotechnology Co., Ltd., Wuhan, China). Finally, quantitative PCR analysis was performed using the 2X Universal SYBR Green Fast qPCR Mix (ABclonal Biotechnology Co., Ltd., Wuhan, China). The primer sequences are shown in Table 2.

### 4.12. Molecular Docking

The Keap1 protein (2FLU) PDB file was downloaded from the RCSB website (https://www.rcsb.org/) (accessed on 5 December 2023) [51]. The protein was dehydrated, and all hydrogen atoms were added before exporting it in PDBQT format for docking analysis. Use the software to draw the three-dimensional structure of a small molecule and add hydrogen atoms. The molecules were set as ligands, and torsion bonds and central structures were checked for rationality before exporting in PDBQT format. A docking box was defined using Autodock, and the GPF file was generated. Autogrid 4 was run with appropriate parameters, followed by Autodock 4 for docking calculations. Results were visualized using the visualize function in Discover Studio software (version 2019).

### 4.13. Data Analysis

The optical density of cell—stained areas was analyzed using ImageJ software (version win64). Statistical analysis was performed using SPSS software (version 26.0). All data are presented as mean ± standard deviation (SD). One-way analysis of variance (ANOVA) was used for significance testing, with a significance level of *p* < 0.05. GraphPad Prism software (version 10.1.2) was employed for data visualization and graphing.

## 5. Conclusions

This study systematically evaluated the protective effects of S1, S5, S6, and S7 on CSE-induced MLE-12 cells. Treatment with S1, S5, S6 and S7 significantly restored the cell viability of MLE-12 cells damaged by CSE induction, and enhanced cellular antioxidant capacity by increasing GSH content and the activities of SOD and CAT, while reducing ROS and MDA levels. S1, S5, S6 and S7 decreased the intracellular levels of the inflammatory factors IL-1β, IL-6 and TNF-α, thereby alleviating cellular inflammatory responses. In addition, S1, S5, S6 and S7 exerted protective effects against CSE-induced mitochondrial dysfunction, reduced the apoptosis rate of MLE-12 cells, significantly promoted cell migration, and accelerated wound closure in a concentration-dependent manner. The cytoprotective mechanisms of S1, S5, S6, and S7 involve enhancing the cellular antioxidant and anti-inflammatory capacities by activating the Keap1/Nrf2 and NF-κB signaling pathways, regulating Bcl-2/Bax expression to inhibit apoptosis, and promoting cell migration to enhance cellular repair functions. This study provides a theoretical basis for the use of S1, S5, S6, and S7 as therapeutic agents in health products for the treatment of cigarette smoke-induced COPD. However, the current research is limited to in vitro cell models. To further validate the functional properties of S1, S5, S6, and S7 as nutritional supplements or health food ingredients in vivo, additional scientific investigations are required.

## Figures and Tables

**Table 1:** Molecular docking results of tuna peptide and Keap1 protein.

Peptide	Binding Energy (Kcal/mol)	Hydrogen Bond Interaction Point	Hydrophobic Interaction Point	Other
S1 (DVGRG)	−7.09	Tyr525, Ser602, Ser363, Asn382, Arg380	Tyr334	Gly364, Arg415
S5 (PHPR)	−7.45	Gly558, Ile559, Leu365, Val604, Val465, Gly367, Val606, Val418	Ala607	—
S6 (GRVPR)	−7.48	Arg415, Gly603, Val463, Ile416, Ser555, Ser602	Phe577	—
S7 (SVTEV)	−3.81	Gly603, Arg380, Arg415, Gln530	Tyr525, Ala556	—

**Table 2:** Primer sequences.

Primer Name	Primer Sequence
Keap1-F	AAAATCATTAACCTCCCTGTTGAT
Keap1-R	CGGCGACTTTATTCTTACCTCTC
Nrf2-F	TTCCCGGTCACATCGAGAG
Nrf2-R	TCCTGTTGCATACCGTCTAAATC
GAPDH-F	GGAGCGAGATCCCTCCAAAAT
GAPDH-R	GGCTGTTGTCATACTTCTCATGG
NfκB-P65-F	CACGAATGACAGAGGCGTGTA
NfκB-P65-R	GCTGCTTGGCGGATTAGCT
IκBα-F	CTGTCCCTGAACCCTATGA
IκBα-R	ACCAGCCAGACCTTGAATA
Bax-F	AGACAGGGGCCTTTTTGCTAC
Bax-R	AATTCGCCGGAGACACTCG
Bcl-F	GAGCCTGTGAGAGACGTGG
Bcl-R	CGAGTCTGTGTATAGCAATCCCA

## Data Availability

The authors declare that the supporting data of this study are available within the article.
